# Comparison of metamizole and paracetamol effects on colonic anastomosis and fibroblast activities in Wistar rats

**DOI:** 10.1186/s40360-020-0383-x

**Published:** 2020-01-13

**Authors:** Eko Purnomo, Dwi Aris Agung Nugrahaningsih, Nunik Agustriani, Gunadi

**Affiliations:** 1 Pediatric Surgery Division, Department of Surgery, Faculty of Medicine, Public Health and Nursing, Universitas Gadjah Mada/UGM Academic Hospital, Yogyakarta, 55291 Indonesia; 2 Department of Pharmacology and Therapy, Faculty of Medicine, Public Health and Nursing, Universitas Gadjah Mada, Yogyakarta, 55281 Indonesia; 3 Pediatric Surgery Division, Department of Surgery, Faculty of Medicine, Public Health and Nursing, Universitas Gadjah Mada, Yogyakarta, 55281 Indonesia; 4 Pediatric Surgery Division, Department of Surgery, Faculty of Medicine, Public Health and Nursing, Universitas Gadjah Mada/Dr. Sardjito Hospital, Yogyakarta, 55281 Indonesia

**Keywords:** Colonic anastomosis, Fibroblast activities, Metamizole, Paracetamol, Wistar rat

## Abstract

**Background:**

Leakage following colorectal anastomosis surgery causes various complications associated with high morbidity and mortality, especially in pediatric patients. It might be caused by the use of non-steroidal anti-inflammatory drugs (NSAIDs) as postoperative analgesics. This study aimed to compare the effect of metamizole and paracetamol on colonic anastomosis and fibroblast activities, including proliferation, migration, and collagen synthesis, in Wistar rats.

**Methods:**

Rats were divided into control, paracetamol and metamizole groups. The colonic anastomosis was evaluated by determining the integrity of the muscle layers, the formation of granulation tissue, and mucosal anastomosis. Fibroblast activities were analyzed by measuring the proliferation, migration, and collagen synthesis.

**Results:**

Metamizole caused more damage to muscle layer integrity, more inhibition of granulation tissue formation in the anastomosis area and lower mucosal anastomosis compared with paracetamol and control groups. Metamizole had a higher cytotoxic effect than paracetamol, which suppressed the proliferation and migration of fibroblasts. Furthermore, both drugs did not affect the synthesis of collagen.

**Conclusion:**

Metamizole shows worse effects on the integrity of muscle layers, inhibition of granulation tissue formation, mucosal anastomosis, fibroblast proliferation, and migration, but not collagen synthesis, than paracetamol in Wistar rat intestines following colonic anastomosis. These findings might indicate that paracetamol is safer than metamizole as analgesic following colonic anastomosis.

## Background

Anastomotic leakage is the most serious complication of colorectal surgery that significantly increases the morbidity and mortality rate of the patients [1]. Anastomosis failure after gastrointestinal surgery is still high ranging between 1.8–19% and there has been no decline over the past 2 decades [2]. In cases of pediatric surgery, 1.3–2.9% of anastomotic leakage cases involved patients who underwent stoma closure surgery [3, 4].

Many factors contribute to anastomotic leakage, including the use of non-steroidal anti-inflammatory drugs (NSAIDs) as postoperative analgesics [5]. An antiinflammatory effect of those analgesics has been suggested through inhibition of cyclooxygenase (COX) isoenzymes activity. Cyclooxygenase converts arachidonic acid into prostaglandins, prostacyclin, and thromboxane. The inflammatory stage leads the early step of wound healing which related with various important cascades during wound repair process. Alteration of the COX isoenzymes activity might interfere with inflammation and wound healing. Giving NSAIDs can indirectly interfere with the healing process of anastomosis by inhibiting the inflammatory process as part of the initial healing process [6, 7]. Although it is useful for all surgical patients, some studies in animals and some clinical data showed detrimental effects of NSAIDs on intestinal anastomosis by increasing the risk of anastomotic leakage [5]. Inflammation is the body’s reaction process, needed to speed up the process of wound healing through the infiltration of fibroblasts, blood vessel growth, fibroblast migration, phagocytosis and collagenation by fibroblasts in the anastomosis region. Fibroblasts are essential cells that are important in the wound healing process. Fibroblasts are found in the intestinal tissue which become active after anastomotic surgery to activate other cells in the wound healing process [6].

NSAIDs, particularly metamizole and paracetamol, are widely used as analgesic therapy after surgery in pediatric cases [8]. However, the effects of metamizole and paracetamol on the safety of colonic anastomosis are controversial. Furthermore, the impact of metamizole and paracetamol on fibroblast activities is still unclear. Therefore, this study aimed to compare the effect of metamizole and paracetamol on colonic anastomosis and fibroblast activities, including proliferation, migration, and collagen synthesis, in Wistar rats.

## Methods

### Subjects

For in vivo studies, we used 3-month-old Wistar rats with body weight of 250–300 g. Rats were obtained from the Department of Pharmacology and Therapy Faculty of Medicine, Public Health and Nursing Universitas Gadjah Mada, Indonesia. Our research protocol referred to the provisions of the principles of handling experimental animals and has obtained ethical permission about research using experimental animals from our institution’s ethics commission. All animals in our study were maintain in international standard animal facility in the best possible conditions and got the best possible care from skilled and experienced animal caregiver. They were acclimatized for 7 days with controlled room temperature and received a regular 12/12 h lighting cycle. Experimental animals were given standard feed and water ad libitum. For the in vitro studies, fibroblasts were primary isolated from the colons of healthy Wistar rats.

### Treatment

Rats were divided into 3 groups of 6 rats each. All groups underwent intestinal anastomosis surgery. After the operation, each group received a different analgesic therapy. One group served as the control which only received aquadest therapy, while the other two groups received either metamizole therapy (60 mg/kg/day) or paracetamol (60 mg/kg/day) as previous study [9]. For in vitro studies, rat colon fibroblasts were cultured with the number of cells each of 1.75 × 105 and divided into 3 groups, which were the control, metamizole and paracetamol groups with 3 different doses each (250 μg/mL, 50 μg/mL, and 5 μg/mL).

### Operating procedure

Anastomotic operations were conducted under sterile conditions. Rats were anesthetized using intramuscular anesthesia containing 0.5 ml ketamine (100 mg/ml), 0.125 ml xylazine (20 mg/ml), 0.075 ml acepromazine (10 mg/ml) and 3 ml sterile saline at 0.1 ml/100 g body weight. All rats received midline 2 cm laparotomy followed by 0.5 cm intestine resection. All resections were performed to intestinal section 5 cm distal from the caecum. The intestinal connection was done with end to end anastomosis with all 5–8 layers inverted, with interrupted sutures. The abdominal wall was closed by simple interrupted suture. After the operation, each rat received an analgesic according to the group: control, paracetamol, and metamizole. After 3 days post-operation, rats were euthanized using high dose of anesthesia containing ketamine, xylazine, and acepromazine at 3 times higher than normal dose (0.3 ml/100 g body weight). We proceeded to sacrifice the rats until no heart beat was detected for at least 5 min. Furthermore, the intestinal segment with anastomosis was removed for further examination.

### Intestinal anastomosis assessment

The intestinal anastomosis was assessed by scoring the integrity of muscle tissue, granulation tissue, and mucous anastomosis. The examination was conducted on histological preparations. The intestines with anastomosis were made into paraffin blocks then hematoxylin-eosin stained to determine sample histology in general. The integrity of the colon muscle tissue and the mucosal anastomosis were assessed and scored. Granulation tissue was assessed by the infiltration of inflammatory cells in the anastomosis area.

### Fibroblast activities measurement

Activation of fibroblasts was assessed from proliferation, migration, and synthesis of collagen. Proliferation was assessed by comparing the level of IC50. Migration was assessed by calculating the difference between before and after injuring fibroblast cultures with the scratch assay method. Collagen synthesis was determined by calculating the absorbance of Sirius red staining on fibroblasts.

### Fibroblast migration test with scratch wound assay

Wounding on cultured cells was done by scraping fibroblast cells in each well using 10–200 μL tip pipettes or blue micropipettes then incubating at 37 °C, 10% CO2 for 1 × 24 hours. After incubation, the well was washed with PBS twice and 500 μL of Meyer hematoxylin were added in each well then incubated at room temperature for about 1 min. Furthermore, each well was filled with 1 mL phosphate buffered saline (PBS), then microscopic images were converted to JPEG format, and empty space pixels and white pixels were calculated with ImageJ software.

### Fibroblast cell proliferation test

Cells were incubated for 24 h. Next, the appropriate treatment for each well was added: 0.9% saline or paracetamol (concentration 250 μg/mL, 50 μg/mL, or 5 μg/mL) or metamizole (concentration 250 μg/mL, 50 μg/mL, or 5 μg/mL). The control group received sterile aquadest treatment. The cells were incubated again for a specified time, which was 48 h. The culture media in each well were transferred into microtubes. Pepsin was added to each well and incubated for 10 min, then the solution from each well was transferred to the appropriate microtube. For each cell suspension microtube, 5 μL cell suspension was taken and then mixed with 5 μL trypan blue. The number of cells in the mixture was calculated using a counting chamber under a microscope.

Each treatment with NSAIDs was done on a triplicate basis and repeated three times. After 48 h of incubation, the numbers of live and dead fibroblasts in each treatment and control group were calculated. Afterward, the percentage of cell death in each well was calculated then converted to probit value. Next, a linear regression equation was made between the log concentration and the probit value so that the IC50 value was obtained as an antilog from the point where y = 5. After that, the average IC50 of each NSAIDs type was calculated.

### Collagen synthesis test

Cells were given each treatment for 24 h, then the media was aspirated and washed with 200 μL PBS 3 times per well. After that, the well was fixed with a 100 μL Bovine solution for 1 h. Then, the well was washed with distilled water until clean and dried overnight. Next, a solution of 100 μL of Sirius Red was given in each well and incubated for 1 h. Next, the Sirius Red dye was removed and the well was washed with 100 μL 0.1 N HCl for 2–3 times. Then, the HCl was removed and washed until Sirius Red’s solution was cleared. 200 μL of 0.5 N NaOH was added to the well and was left for 30 min. The absorbance reading was conducted at a wavelength of 570 nm with a plate reader.

### Data analysis

Data analysis was conducted using the SPSS Statistics 17.0 for Windows application. The data obtained were tested for normality in advance by the Kolmogorov Smirnov test to determine whether the data was normally distributed. If the data were normally distributed, the student’s t-test was done, and if they were not normally distributed, the Mann-Whitney U-test was used.

## Results

### In vivo metamizole and paracetamol effect on intestinal anastomosis

**Fig. 1:**
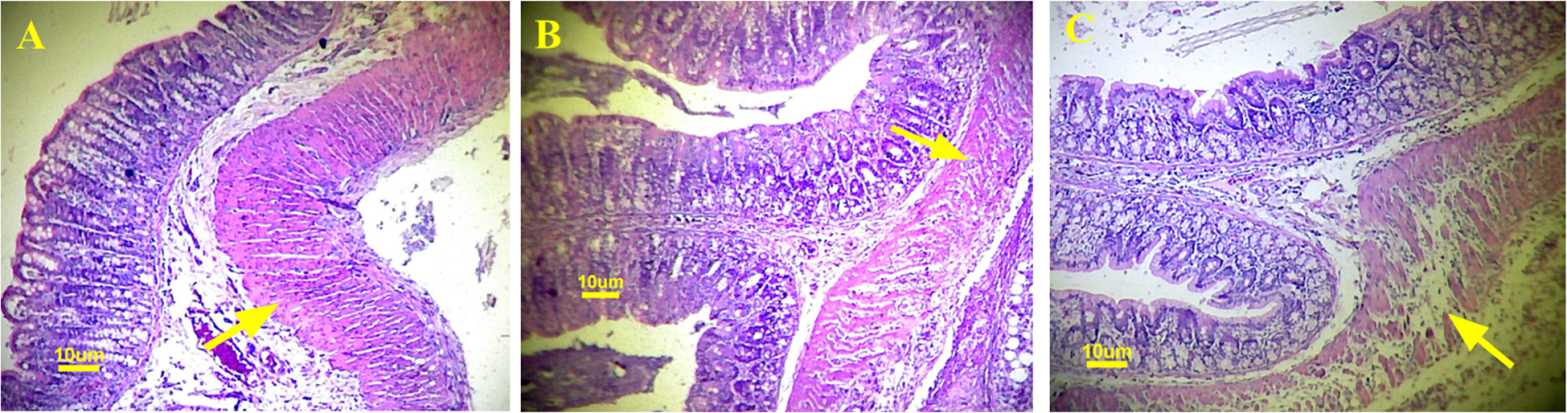
Colonic histology of anastomosis in each treatment group (**a**. Control, **b**. Paracetamol, **c**. Metamizole). The structure of the serous, submucosal and mucosal layers in anastomosis area is separated in the metamizole group, whereas in paracetamol and control groups, the structure of intestinal tissue is better

**Fig. 2:**
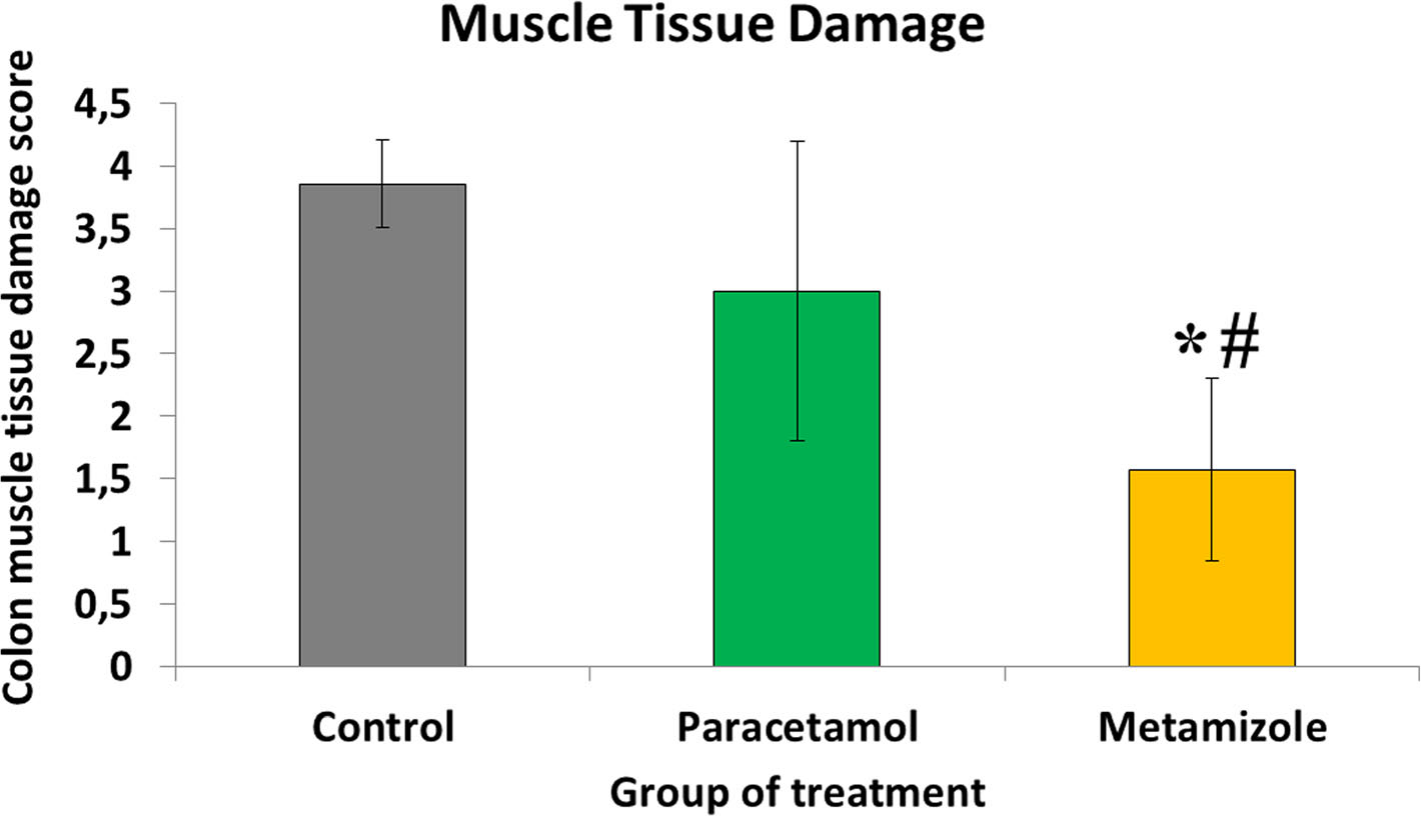
Scoring of colon muscle layer damage in anastomosis area (*, metamizole vs. control, *p* < 0.05; #, metamizole vs. paracetamol, *p* < 0.05). Low values indicating heavier damage

Histopathological findings showed more anastomosis failure in the metamizole group compared with the paracetamol and control groups **(**Fig. 1**)** Damage to the muscle layers as well as inflammatory tissue and granulation which occurred in the anastomosis area were assessed using the scoring system from histological samples **(**Fig. 2**)**. It was found that muscle damage in the colonic anastomosis area was more severe in the metamizole group (1.57 ± 0.8) compared to paracetamol (3 ± 1.3) and control (3.86 ± 0.38) with a value of *p* < 0.05. Paracetamol did not have negative effects of colon muscle damage during the anastomosis process compared to controls (*p* > 0.05).

**Fig. 3:**
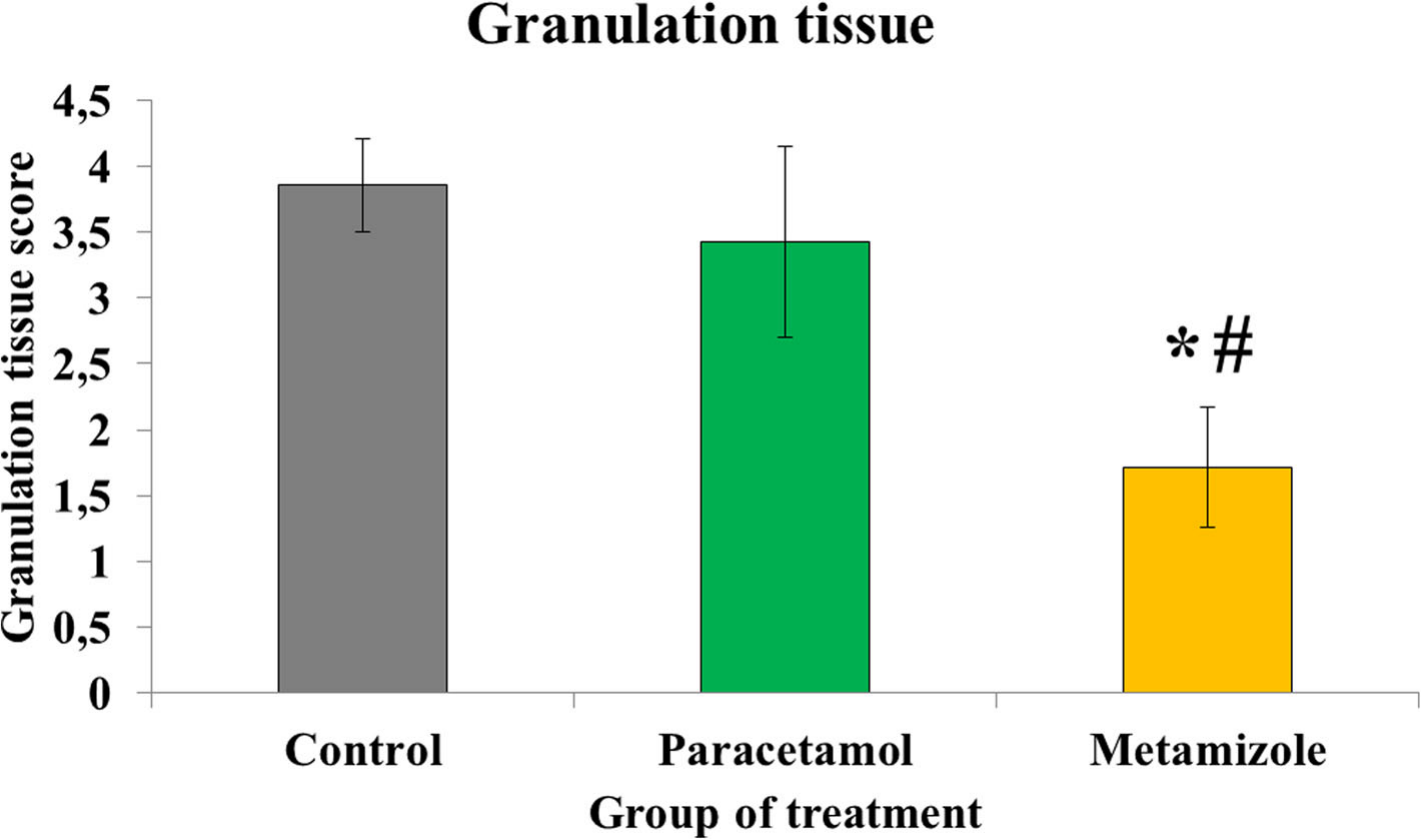
Scoring of granulation tissue on the anastomosis side of rat colon (*, metamizole vs. control, *p* < 0.05; #, metamizole vs. paracetamol, *p* < 0.05). Low values indicating worse granulation tissue

Granulation tissue formation on anastomosis colon was more inhibited in the metamizole group (1.71 ± 0.5) than paracetamol (3.43 ± 0.8) and control groups (3.86 ± 0.4) (*p* < 0.05). In addition, the granulation tissue formation was comparable between paracetamol and control groups (*p* > 0.05) **(**Fig. 3).

**Fig. 4:**
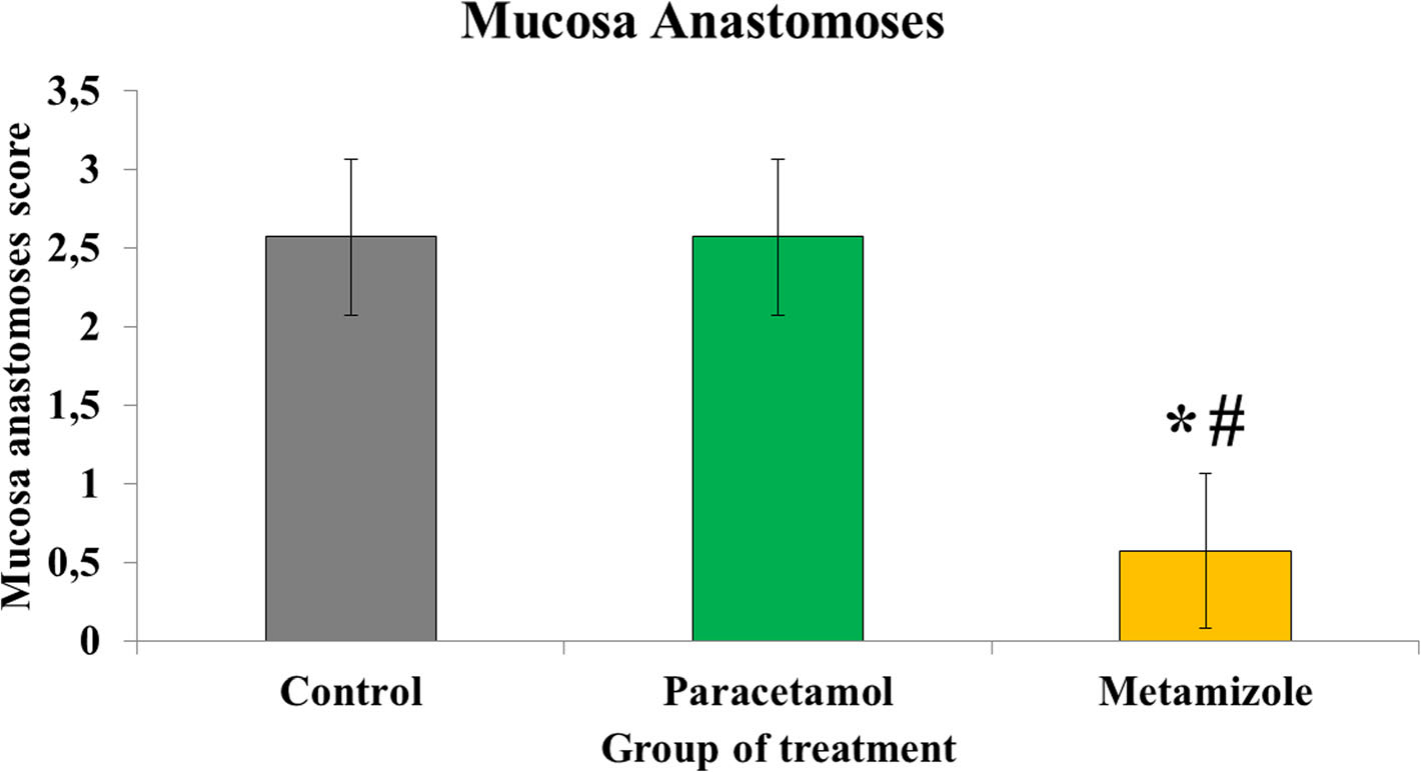
Scoring of anastomosis of colon mucosa in the anastomosis area (*, metamizole vs. control, *p* < 0.05; #, metamizole vs. paracetamol, *p* < 0.05)

The average of mucosal anastomosis in the metamizole group was lower (0.57 ± 0.5) when compared with both paracetamol (2.57 ± 0.5) and control (2.57 ± 0.5) groups with a *p*-value of < 0.05. Furthermore, the level of mucosal anastomosis was similar between the paracetamol and control groups (*p* > 0.05) **(**Fig. 4**)**.

### In vitro impact of metamizole and paracetamol on fibroblast activities following colonic anastomosis

**Fig. 5:**
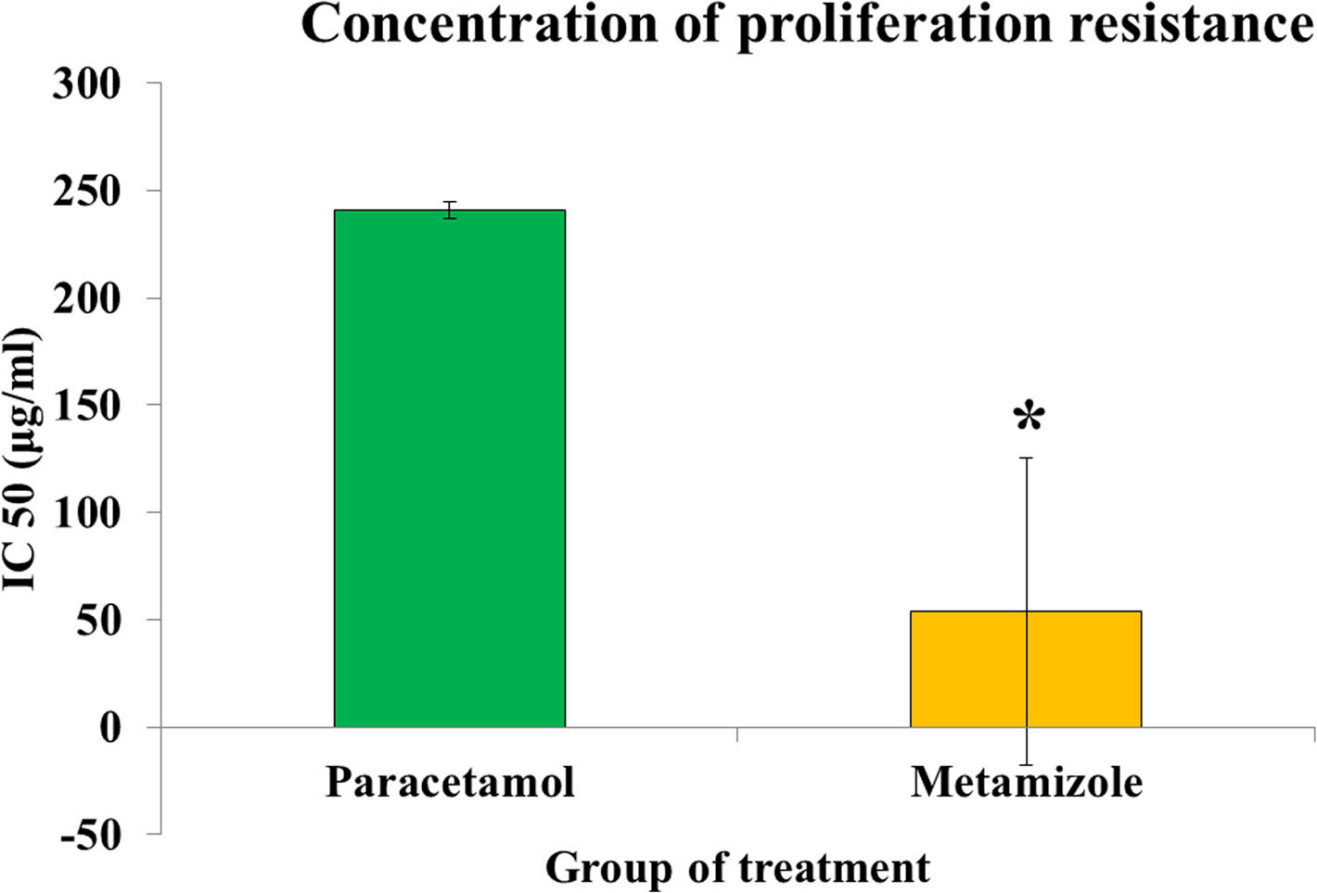
Proliferation resistance concentration of paracetamol and metamizole on rat colon fibroblast cells. Metamizole is more cytotoxic than paracetamol (*, *p* < 0.05)

Metamizole has significantly lower IC50 value compared to paracetamol (53.9 ± 75.9 vs. 240.7 ± 4.1 μg/mL; *p* < 0.05), indicating metamizole has a more toxic effect in inhibiting fibroblast proliferation compared to paracetamol **(**Fig. 5**)**.

**Fig. 6:**
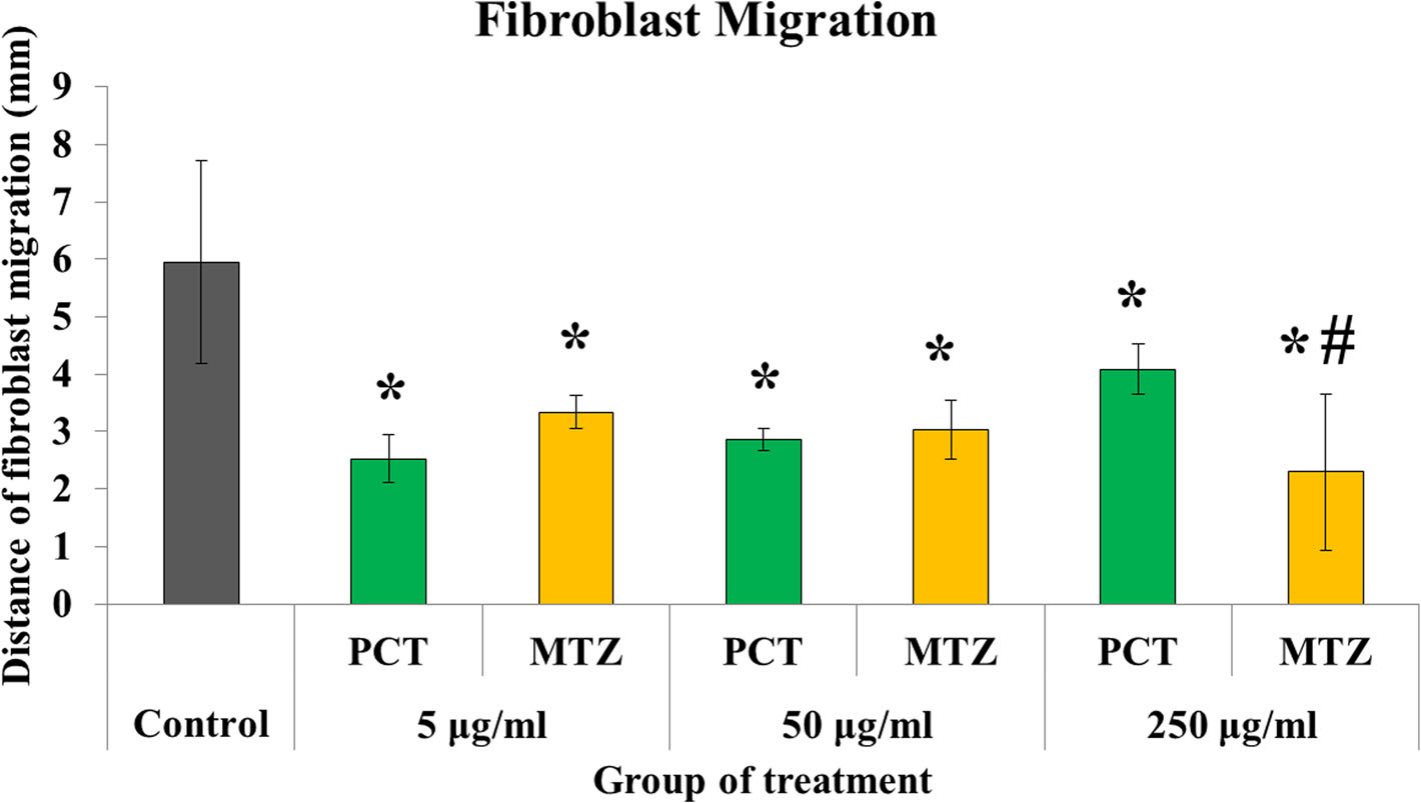
Average of fibroblast migration activity with various treatments for 24 h (PCT, paracetamol; MTZ, metamizole; *, PCT or MTZ vs. control, *p* < 0.05; #, PCT vs. MTZ, *p* < 0.05)

**Fig. 7:**
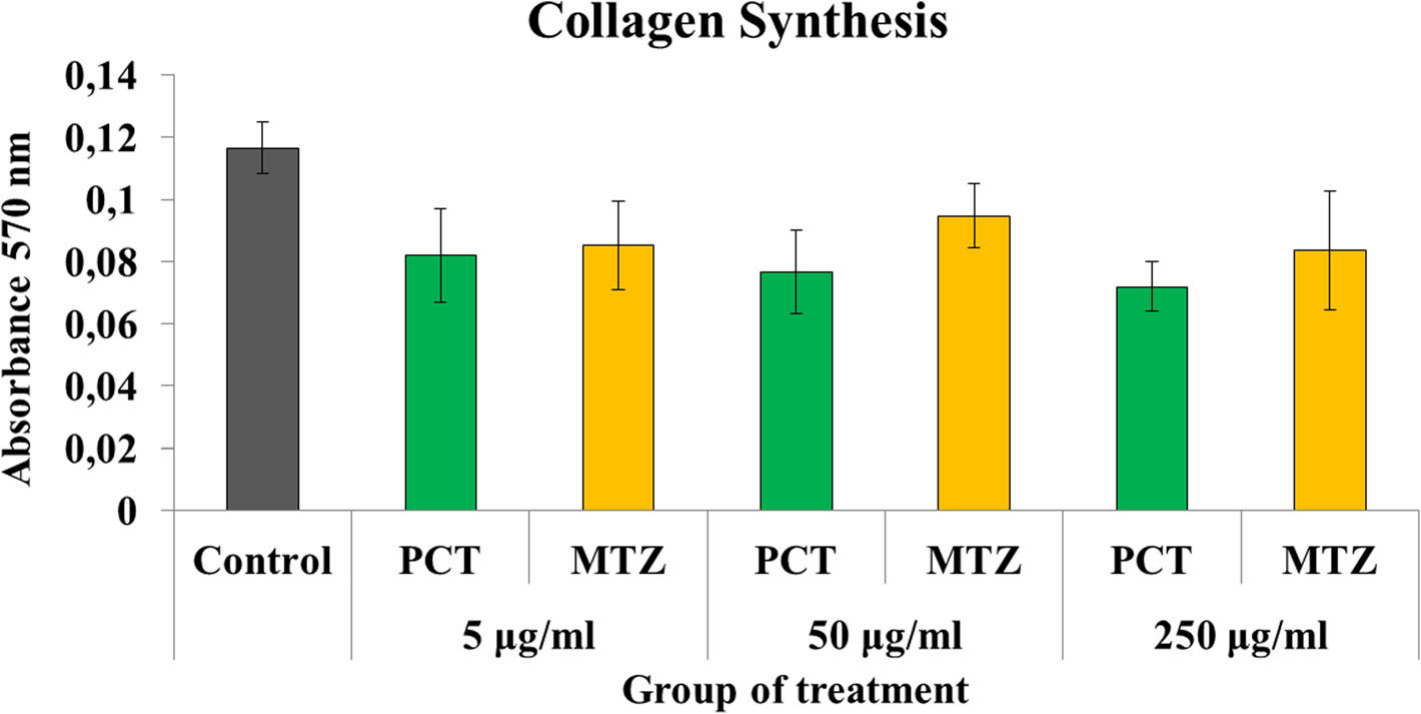
Average collagen synthesis activity in fibroblast with various treatments. There is no difference between the three groups that inhibits collagen synthesis (*p* > 0.05)

The fibroblast migration was inhibited more by paracetamol and metamizole compared with control group (*p* < 0.05) **(**Fig. 6**)**. The inhibition of metamizole and paracetamol on the migration of fibroblast was equivalent at doses of 5 μg/ml (2.34 ± 0.29 vs. 2.53 ± 0.42 mm; *p* > 0.05) and 50 μg/ml (3.04 ± 0.51 vs. 2.86 ± 0.20 mm; *p* > 0.05), but the effect of metamizole was higher in suppressing migration activity than paracetamol at the higher dose concentration of 250 μg/ml (1.92 ± 1.11 vs. 4.08 ± 0.44 mm; *p* < 0.05). Furthermore, paracetamol and metamizole did not affect the synthesis of colon fibroblast collagen in the 48 h after surgery compared with the control group at all treatment doses (*p* > 0.05) **(**Fig. 7**)**.

## Discussion

In this study, results shown that metamizole has a worse effect than paracetamol on colonic anastomosis. Metamizole also had higher antiproliferative and antimigration effects on colon fibroblasts, but not collagen synthesis, than paracetamol. To the best of our knowledge, our study is the first report of comparison between metamizole and paracetamol on the colonic anastomosis. Another novelty of our study is we showed the impact of metamizole and paracetamol on the fibroblast activities.

The role of the inflammatory process is very important in healing anastomosis wounds which are characterized by the formation of granulation tissue [7]. Our in vivo studies showed that metamizole inhibited the anastomosis process of rat colons. These results were in accordance with the previous study that found metamizole tends to increase the incidence of anastomotic leakage [10]. In contrast, there was no significant inhibition in the anastomosis process of rat colon in the paracetamol group, which was supported by previous reports [11, 12].

We also showed that metamizole has a negative influence on the integration of the muscle layers of the rat colon wall. It might be related to the direct mechanism of non-selective resistance to COX-1 and COX-2 enzymes. If the enzyme activity was inhibited, then it will affect prostaglandin synthesis which is an important mediator in the inflammatory process [13].

In addition, metamizole also suppressed the process of granulation tissue formation in the anastomosis site. The inhibition of cyclooxygenase enzymes by metamizole might reduce prostaglandin synthesis which affected the process of granulation tissue formation by inhibiting vasodilation of blood vessels at the site of the wound so that the leukocyte migration process was reduced. There was also a decrease in leukocyte proliferation in the inflammatory area [13].

Our findings also revealed metamizole inhibited mucosal anastomosis healing, while paracetamol did not affect the process. These results were consistent with data from previous studies where the strength of rat colonic anastomosis joints was not affected by the administration of both low and high doses of paracetamol [11]. This may be due to the central effect of paracetamol which was more dominant than peripheral effect in inhibiting prostaglandin synthesis [11].

Our in vitro findings revealed metamizole was more cytotoxic to fibroblasts compared to paracetamol. It has been reported that paracetamol, which has mild anti-inflammatory effect [14], requires a larger dose to obtain the same proliferative inhibitory power as metamizole which has a higher anti-inflammatory effect [15]. In addition to the IC50 value of metamizole and paracetamol, we also needed to know the maximum concentration (Cmax) of these drugs. The administration of 1 g of metamizole intravenously will obtain a value of Cmax 56.5 μg/mL [16]. Whereas the same dose of paracetamol will only produce Cmax 19–22 μg/mL [17, 18]. When comparing the IC50 with Cmax of each treatment, the IC50 metamizole (53.9 ± 75.9 μg/mL) value was below the Cmax so that the inhibition concentration could easily be reached in the blood. However, this does not apply to paracetamol, where the IC50 value (240.7 ± 4.1 μg/mL) was above the Cmax so it will be difficult to achieve inhibition concentration if the drug was given in therapeutic doses. Therefore, the administration of paracetamol in therapeutic doses was very unlikely to give an adverse effect of anti-inflammation as can be caused by metamizole. This proliferation barrier was consistent with previous studies [19, 20] about the antiproliferative effects of NSAIDs on rat and human fibroblasts. The antiproliferation effects of NSAIDs occur in direct barriers to the increase of cyclooxygenase enzymes in the inflammatory process [20, 21]. The inflammatory response will activate the COX-2 enzyme thereby increasing the synthesis of PGE2 which can inhibit proliferation of fibroblasts [21–23]. The antiproliferative effects of NSAIDs are also accompanied by barriers to DNA synthesis [18]. Metamizole which has a cyclooxygenase non-selective inhibitor action will suppress COX-2 enzyme activation so that it can suppress the DNA synthesis process and proliferation of rat colon fibroblasts. Metamizole has a more potent antiproliferative effect in the pancreatic cell line, Panc-1, than paracetamol at the highest dose concentration of 250 μg/ml [24].

The effect of metamizole inhibition was also seen in the migration of fibroblasts. This inhibition effect of metamizole was dose-dependent, and appears to be more dominant than paracetamol at the highest dose of treatment. According to Nicpon et al. [25], the effect of metamizole inhibition on cell function is dose-dependent on the concentration. The higher the concentration, then more obstacles will occur. Our results showed similar results where the inhibition of fibroblast migration increased with increasing metamizole concentration. Paracetamol appeared to also have a negative effect on fibroblast migration activities. Even so, the effect did not increase with the addition of the treatment dose. The inhibition of fibroblast migration by paracetamol was still less compared to metamizole at the highest dose of 250 μg/ml. These results were in accordance with previous studies which showed that paracetamol was one of the NSAIDs which has the lowest anti-inflammatory effect. The inhibition of fibroblast migration by NSAIDs is to suppress the action of the cyclooxygenase enzyme. These effects can be restored by the administration of exogenous prostaglandins [26]. In addition, the mechanism for the inhibition of fibroblast migration is through the matrix metalloproteinase enzyme pathway known as RECK (reversion-inducing-cysteine-rich protein with Kazal motifs) [27].

The activity of fibroblasts in synthesizing collagen will increase in the stages of anastomosis wound healing. However, our study showed that metamizole and paracetamol did not reveal any significant inhibitions of collagen synthesis compared to control. In addition, previous in vivo reports revealed that barriers due to metamizole and paracetamol to collagen synthesis are equivalent to the control group [28, 29].

It should be noted that the effect of NSAIDs on the activity of fibroblasts has not been able to explain the whole process of intestinal anastomosis, because of the important role of other cells such as mucosal epithelial cells, smooth muscle cells lining the intestinal wall, intestinal endothelial vessels, and various inflammatory cells in the healing process of intestinal anastomosis wounds. In addition, in this study NSAIDs treatment was given under normal fibroblast conditions so that it might be different from the inflammatory conditions in the wound healing process.

Further study is necessary to clarify the inhibitory and migratory effects of metamizole and paracetamol on cyclooxygenase enzymes in rat and human fibroblast cells. Using in vitro methods with co-culture techniques will provide better understanding of the effects of NSAIDs on the interactions between inflammatory cells and mucosal, endothelial and fibroblast epithelial cells in the process of wound healing.

We conducted this study based on the clinical data that showed a detrimental effect of NSAIDs on colon anastomosis. Thus, we investigated both NSAIDs, metamizole and paracetamol, that widely used as post-surgery analgesia in pediatric patients to compare their effects on rat colonic anastomosis. Based on our in vivo and in vitro results, we believe that this study will give more evidence to improve post operative analgesic management in pediatric patients.

## Conclusion

Metamizole shows worse effects on integrity of muscle layer, inhibition of granulation tissue formation, mucosal anastomosis, fibroblast proliferation, and migration, but not collagen synthesis, than paracetamol in Wistar rat intestines following colonic anastomosis. These findings might indicate that paracetamol is safer than metamizole as an analgesic following colonic anastomosis.

## Data Availability

All data generated or analyzed during this study are included in the submission. The raw data are available from the corresponding author upon reasonable request.

## Abbreviations

Cmax: Maximum concentration
COX: Cyclooxygenase
IC50: Half maximal inhibitory concentration
MTZ: Metamizole
NSAIDs: Non-steroidal anti-inflammatory drugs
PBS: Phosphate-buffered saline
PCT: Paracetamol
RECK: Reversion-inducing-cysteine-rich protein with Kazal motifs
